# Toward Machine-Learning-Accelerated Design of All-Dielectric Magnetophotonic Nanostructures

**DOI:** 10.1021/acsami.4c06740

**Published:** 2024-07-30

**Authors:** William O. F. Carvalho, Marcio Tulio Aiex Taier Filho, Osvaldo N. Oliveira, Jorge Ricardo Mejía-Salazar, Felipe Augusto Pereira de Figueiredo

**Affiliations:** † Sao Carlos Institute of Physics, University of Sao Paulo, CP 369, São Carlos, São Paulo 13560-970, Brazil; ‡ National Institute of Telecommunications (Inatel), Santa Rita do Sapucaí, Minas Gerais 37540-000, Brazil

**Keywords:** all-dielectric, machine-learning-accelerated design, magnetophotonic, neural networks, polynomial regression, TMOKE sensing

## Abstract

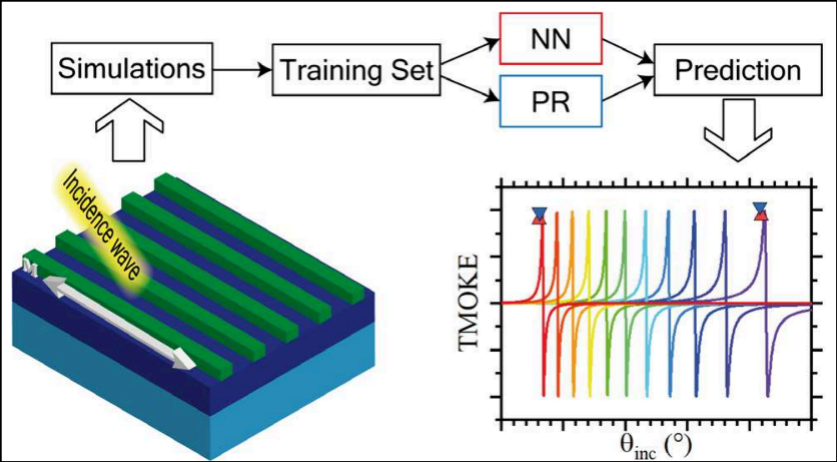

All-dielectric magnetophotonic nanostructures are promising for integrated nanophotonic devices with high resolution and sensitivity, but their design requires computationally demanding electromagnetic simulations evaluated through trial and error. In this paper, we propose a machine-learning approach to accelerate the design of these nanostructures. Using a data set of 12 170 samples containing four geometric parameters of the nanostructure and the incidence wavelength, trained neural network and polynomial regression algorithms were capable of predicting the amplitude of the transverse magneto-optical Kerr effect (TMOKE) within a time frame of 10^–3^ s and mean square error below 4.2%. With this approach, one can readily identify nanostructures suitable for sensing at ultralow analyte concentrations in aqueous solutions. As a proof of principle, we used the machine-learning models to determine the sensitivity (*S* = |Δθ^res^/Δ*n*_a_|) of a nanophotonic grating, which is competitive with state-of-the-art systems and exhibits a figure of merit of 672 RIU^–1^. Furthermore, researchers can use the predictions of TMOKE peaks generated by the algorithms to assess the suitability for experimental setups, adding a layer of utility to the machine-learning methodology.

## Introduction

Developing a new host of active optical devices depends upon the ability to design nanostructures whose optical properties can be modulated dynamically. In recent years, the optical response of magnetic materials has been tuned by exploiting magneto-optical (MO) effects,^1^ which has led to breakthroughs in (bio)sensing,^1^ routers/switches,^2^ modulators,^3,4^ circulators and isolators,^5−7^ and buffering.^8^ Because MO effects are weak in the nanoscale, enhancement has been sought through plasmonic resonance incorporating metallic building components. MO activity is increased in these magnetoplasmonic (MO metal) nanostructures that have enhanced, localized plasmonic fields (at the metal surface) distributed inside an adjacent MO layer.^9^ However, energy efficiency is limited by the intrinsic joule heating in these magnetoplasmonic systems. This has motivated the use of all-dielectric magnetophotonic nanostructures,^10−12^ which feature low levels of losses as a result of the lack of joule heating. The main challenge in designing these all-dielectric nanophotonic platforms is to identify the optical resonances with high MO amplitudes. Another difficulty is related to the confinement of optical fields within the scatterers with the highest refractive index (hindering interaction with nearby MO materials), in contrast to magnetoplasmonic approaches, where the field is confined at metal/dielectric surfaces.^9^ Owing to such problems, current methodologies for designing all-dielectric MO nanostructures require time-consuming electromagnetic simulations, mostly with trial-and-error strategies. Additionally, the design of each nanostructure for a given magnetization state demands a meticulous analysis to attain the desired MO features, making the process laborious and iterative. Strategies are therefore needed to overcome these drawbacks when designing all-dielectric nanostructures with tailored MO properties.

One possible solution is to employ machine-learning (ML) algorithms that could identify nanostructures with optimized values by training with a limited number of electromagnetic simulations. In fact, neural networks (NNs) and other ML methods have proven effective for the design of core–shell nanoparticles^13^ and metasurfaces,^14^ in addition to other examples of material design and discovery that reduced the reliance on labor-intensive experiments and simulations.^15,16^ While some prior works have employed ML techniques for optimizing MO devices, such as MO traps for cold atoms^17^ and MO imaging devices,^18^ this has not been the case for magnetophotonic nanostructures. In this paper, we report on a ML-based approach for the rapid and efficient design of all-dielectric MO nanostructures. Two ML models were used for comparison: NNs and polynomial regression (PR). These models were trained and validated using a data set of 15 213 (comprising 12 170 for training and 3043 for validation) samples to predict the amplitude of the transverse magneto-optical Kerr effect (TMOKE), which can be used in sensing and biosensing. In particular, the algorithms were not specifically trained to deliver designs with targeted TMOKE values. Instead, they were developed to provide researchers with the maximum achievable TMOKE amplitude and sensitivity for a given set of geometrical parameters in a nanostructure. Therefore, our methodology can be applied to various materials and geometries, allowing for broad applications and generalization in nanophotonic-based MO applications.

## Methods and Materials

**Figure 1:**
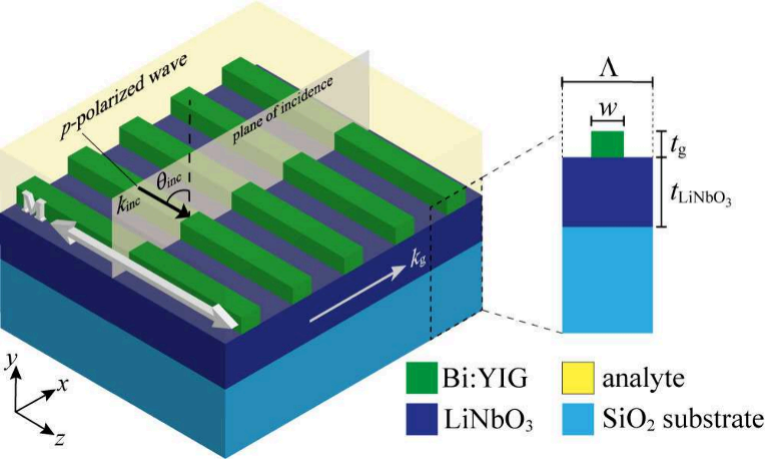
Schematic illustration of all-dielectric magnetophotonic gratings, with the geometric parameters in the inset.

The aim is to automate the design of nanostructures, such as the grating coupler in Figure 1, which comprises a one-dimensional periodic arrangement of dielectric MO ribs placed on a high refractive index (HRI) guiding layer, grown on a SiO_2_ substrate. For a realistic simulation, we considered materials used experimentally, with MO ribs made of bismuth-substituted yttrium iron garnet (Bi:YIG)^19,20^ and the HRI layer made of lithium niobate (LiNbO_3_).^21^ The working wavelength (λ) was varied from 570 to 670 nm, compatible with commercial setups. The corresponding permittivities (as functions of λ) ε_SiO_2__,^22^ ε_LiNbO_3__,^21^ and ε̃_Bi:YIG_^23^
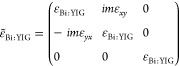
1for SiO_2_, LiNbO_3_, and Bi:YIG, respectively, were used from experimental reports. Because Bi:YIG is an anisotropic material, its permittivity is represented by the tensor ε̃_Bi:YIG_^23^ in eq 1, where ε_Bi:YIG_ and ε_*xy*_ = ε_*yx*_ are the diagonal and off-diagonal tensor components in the transverse configuration (**M**∥*z*, in relation to Figure 1).

Among the different MO configurations (polar, longitudinal, and transverse), we focused on the TMOKE as a result of its unique ability to preserve the polarization of the incident light, because only the amplitude of reflected/transmitted intensity is modulated.^24^ Moreover, the sharp Fano-like curves of TMOKE are used for improved resolution in magnetoplasmonic biosensing and magnetometry,^25,26^ which we also exploit here to illustrate the applicability of our concept. In the transmission mode, TMOKE is defined as^24^
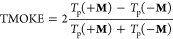
2where *T*_p_(±**M**) is the transmittance amplitude associated with **M** pointing along the ±*z* axis. The superstrate region, referred to as the analyte region in Figure 1, is considered to possess a refractive index *n*_a_ varying between 1.3 and 1.4 to illustrate the potential application in all-dielectric magnetophotonic biosensing platforms. The geometric parameters of the structure are illustrated in Figure 1, where Λ, *w*, and *t*_g_ are the period length, width, and thickness of the grating ribs, whereas *t*_LiNbO_3__ represents the thickness of the HRI layer. The full-wave (FW) numerical simulations were made using the finite element method (FEM) within the commercial software COMSOL Multiphysics. Floquet periodic boundary conditions were set on the lateral boundaries to consider an infinite periodic system along the *x* axis, while perfectly matched layers (PMLs) were used along the *y* boundaries to avoid undesired numerical reflections.

**Figure 2:**
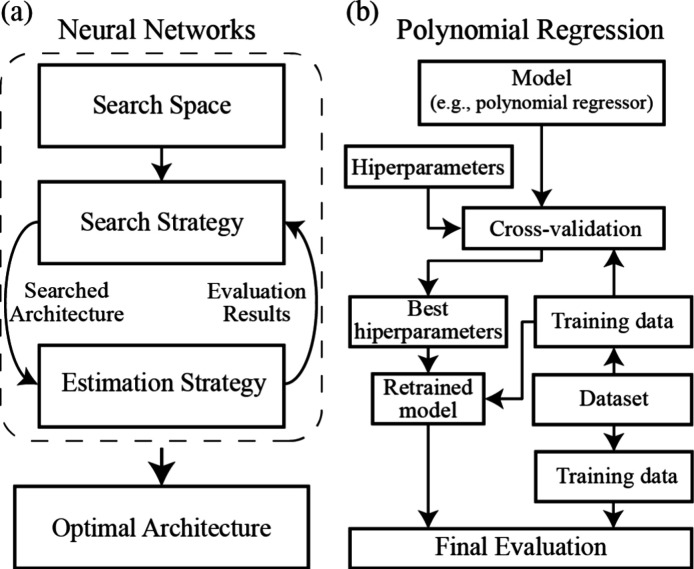
Working flowcharts of (a) NN and (b) PR algorithms.

We compared two ML models for the automated design of the nanostructures, namely, NN and PR. The training and evaluation metric chosen to assess these ML models was the mean square error (MSE). The input data for the ML algorithms consists of a set of geometrical parameters (illustrated in the inset of Figure 1) and the incident wavelength. For computational purposes, the data set has undergone two preprocessing steps. The first step eliminated incoherent and divergent geometrical parameters, also known in the ML context as features. The second step normalized the features to enhance the performance of the ML models. Specifically, the features were normalized to the range [−1, 1]. In contrast, the output of the algorithms corresponds to the maximum TMOKE amplitudes (|TMOKE|) for the refractive indexes 1.3 and 1.4 and their corresponding angles, respectively. Given the input data, supervised training algorithms iteratively minimize the MSE between the expected values, e.g., TMOKE amplitudes (ranging from [0, 2]) and the angles (ranging from [0°, 90°]) corresponding to the selected refractive indexes, and the values predicted by the models. We first used the neural architecture search (NAS) technique to automate searching for the best NN architecture for this specific task.^27,28^ Unlike the tedious and time-consuming trial-and-error process of finding a proper NN architecture, the NAS technique utilizes an optimization algorithm, such as greedy, Bayesian, and hyperband algorithm, to do so.^29^ The evaluation metric chosen at the beginning of the NAS process is MSE. Then, a cyclic optimization algorithm generates candidate NN architectures, which are subsequently trained and assessed on the basis of the evaluation metric.^30^ The latter process is illustrated in the left panel of Figure 2. The search process continues until a termination criterion, such as a trial limit, is reached. The final architecture is the one that demonstrates the best performance according to the evaluation metric. At the end of the search, the optimized NN architecture is evaluated using a set of unseen samples, obtained through FW FEM simulations, to verify its generalization performance.^31^ We used AutoKeras, an open-source NAS library.^31^

The other ML method, PR, is one of the oldest and most efficient ML techniques. It involves adjusting a polynomial function to the data samples, which requires finding the optimal polynomial degree. Cross-validation approaches can be used to discover the best values for hyperparameters, such as the degree of a polynomial function that can be applied to the problem being addressed.^32^ They assess the performance of a model accurately by splitting the data set into multiple training and testing sets, allowing the model to be trained and tested multiple times. Cross-validation avoids the model overfitting to the training data set.^33^ The procedure to find the best PR model is illustrated on the right panel of Figure 2; it employs the *k*-fold approach, which is a simple, efficient cross-validation method.^34^ In the *k*-fold method, the data set is divided into *k* subsets or folds where *k* – 1 subsets are used for model training and 1 subset is used for validation. The method iteratively trains models with different polynomial degrees on *k* training sets created out of the *k* – 1 subsets left for training. After each training iteration, the MSE of the model is calculated using the current validation set. This procedure is repeated *k* times, resulting in *k* MSE values. These values are then averaged and used to estimate the variance of the MSE. The averaged MSE and its variance are the metrics used to compare the performance of this model against others. The previous procedure is repeated for each polynomial degree. Then, the MSE and variance values of all models are compared, and the degree resulting in the smallest values is selected as the best one for the PR model. Finally, the best PR model is also evaluated with the set of unseen samples used previously to assess its generalization capacity. This cross-validation procedure ensures a robust model with a suitable trade-off between underfitting and overfitting.^35^

## Results and Discussion

Optimization of all-dielectric magnetophotonic devices is a key requirement to reach responses suitable for applications, such as sensing. Trial-and-error strategies for experimental fabrication of the devices are not an option owing to the number of geometric parameters and materials involved. Computer simulations are then normally employed, but these are costly in terms of computational resources and time. We, therefore, exploit here an accelerated design optimization by combining ML methods and simulations. The data set used for training the ML algorithms comprises 12 170 samples, which are simulated properties of grating couplers as in Figure 1. Each sample is structured as a 1 × 5 array, with four geometrical parameters illustrated in the inset of Figure 1, and the operating wavelength λ. Thus, an individual sample, labeled “data instance”, is represented as data = [Λ*wt*_g_*t*_LiNbO_3__λ], where the corresponding values in the array consist of pseudorandom combinations of values for 215 ≤ Λ ≤ 255 nm, 70 ≤ *w* ≤ 110 nm, 43 ≤ *t*_g_ ≤ 65 nm, 130 ≤ *t*_LiNbO_3__ ≤ 175 nm, and 570 ≤ λ ≤ 670 nm. Because the resonant angle/wavelength in magnetophotonic gratings normally varies linearly with *n*_a_,^11^ calculations of TMOKE were only made for *n*_a_ = 1.3 and 1.4 (i.e., the extreme values in the range). Hence, for each sample, there are two TMOKE values, namely, TMOKE_1_ (for *n*_a_ = 1.3) and TMOKE_2_ (for *n*_a_ = 1.4), and the resonant angle θ^res^ associated with TMOKE peaks (TMOKE^max^), which were obtained using FW electromagnetic simulations by sweeping the incident angle in the range of 0° ≤ θ_inc_ < 90°. The ML models are then trained to predict these four target values, |TMOKE_1_^max^|, |TMOKE_2_^max^|, θ_1_^res^, and θ_2_^res^. The algorithms provide the values for |TMOKE_1_^max^| and |TMOKE_2_^max^|, which range from 0 to 2 (according to eq 2). The values of θ_1_^res^ and θ_2_^res^, ranging from 0° to 90°, are intricately associated with each TMOKE_*i*_^max^ through the resonant physical properties of the respective nanostructures. It is worth noting that the concept presented here is not limited to a particular set of materials, thus enabling its generalization for various combinations of materials and/or geometries.

**Figure 3:**
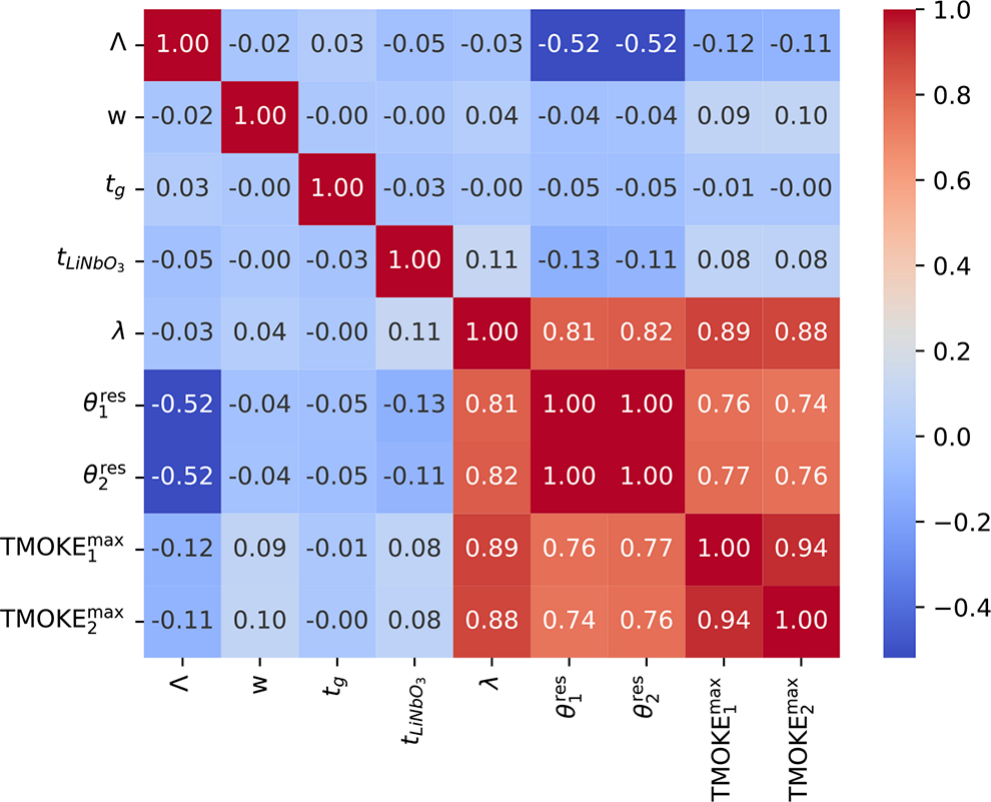
Correlation matrix between each of the input features and target values.

Figure 3 depicts the Pearson correlation coefficient between all pairs of input features and targets. The correlation coefficient refers to the degree to which a pair of variables are linearly related. As seen, there is a high positive correlation (values equal or greater than 0.81) between the λ feature and all of the four targets, |TMOKE_1_^max^|, |TMOKE_2_^max^|, θ_1_^res^, and θ_2_^res^. A positive correlation indicates that, as the attribute value increases, the target values tend to increase. There is also a medium negative correlation of −0.52 between the Λ feature and targets θ_1_^res^ and θ_2_^res^, indicating that, as the attribute value increases, the target value tends to decrease. Apart from that, features *w*, *t*_g_, and *t*_LiNbO_3__ exhibit small to very small correlations with the four targets. However, as some experiments have shown, removing them from the training set has a negative impact on the MSE, increasing it when compared to the scenario where all of them are employed. Therefore, although they do not have a high correlation with the targets, these features might present nonlinear relationships with them that are not accounted for by the (linear) correlation coefficient. If the relationship between two variables is not linear, the correlation coefficient will not fully characterize their relationship. Additionally, because NN and PR models are nonlinear in the sense that they combine input features internally and, in the case of NNs, have nonlinear activation functions, they can explore complex relationships between them and the targets. The figure also shows that the input features are non-co-linear (i.e., the correlation between features is low). Therefore, it is not necessary to remove or create/transform features. Thus, we treat all features equally and employ all of them as input to the PR and NN models.

**Figure 4:**
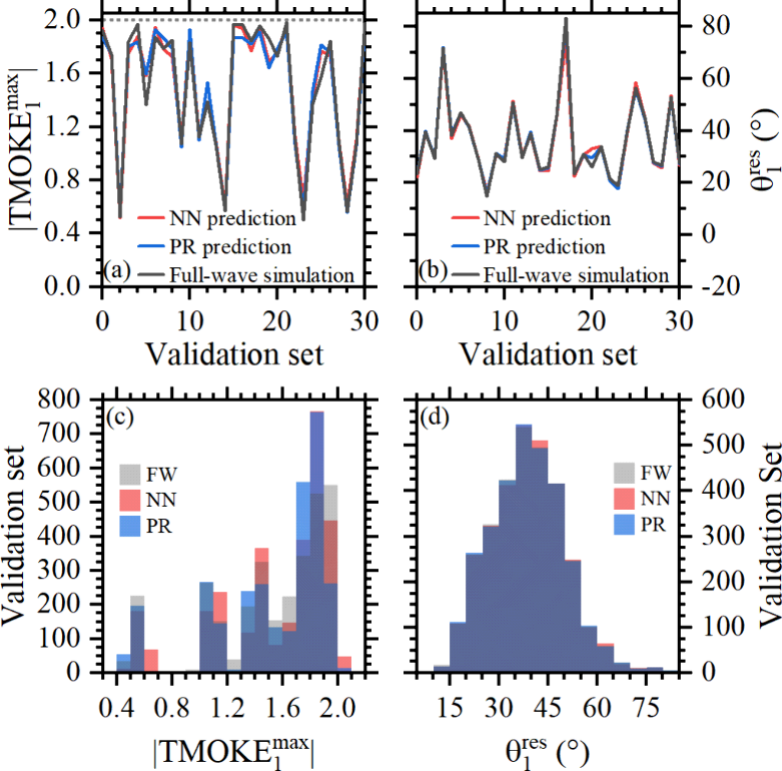
Validation of algorithms: (a) TMOKE_1_^max^ and (b) θ_1_^res^ comparison among FW simulations (black) and NN (red) and PR (blue) predictions and validation histograms for FW simulations (gray), NN (red), and PR (blue) for (c) TMOKE_1_^max^ and (d) θ_1_^res^, respectively.

The most efficient NN and PR models identified had their performance validated using 3043 random samples out of 15 213 of the whole data set. For visualization purposes, Figure 4a shows results only for the first 30 samples in the validation set, as indicated on the horizontal axis. The ML results appear in red and blue for NN and PR, respectively, while the FW numerical simulations are shown in black. Because similar results were obtained for |TMOKE_1_^max^| and |TMOKE_2_^max^| and their corresponding resonant angles θ_1_^res^ and θ_2_^res^, we only show results for the validation samples associated with |TMOKE_1_^max^| and θ_1_^res^ in panels a and b of Figure 4. Small differences for TMOKE_1_^max^ and θ_1_^res^ are observed only in a few validation samples, as can be noted, indicating the feasibility of our approach. Panels c and d of Figure 4 present histograms offering a general overview comparison using all 3043 random validation samples. These histograms compare the distribution of the target values (i.e., results from the numerical simulations) against the distribution of the values predicted by the PR and NN models. As observed, the distributions of both predictions, PR and NN, for θ_1_^res^ almost perfectly match the target distribution. Conversely, the distributions of the predictions for TMOKE_1_^max^ deviate somewhat from the target distribution, with the prediction distribution of the NN model being the closest. This difference might be explained by the fact that the TMOKE_1_^max^ target is highly correlated only with the λ feature, as seen in Figure 3, and the multimodal nature of this distribution.

**Figure 5:**
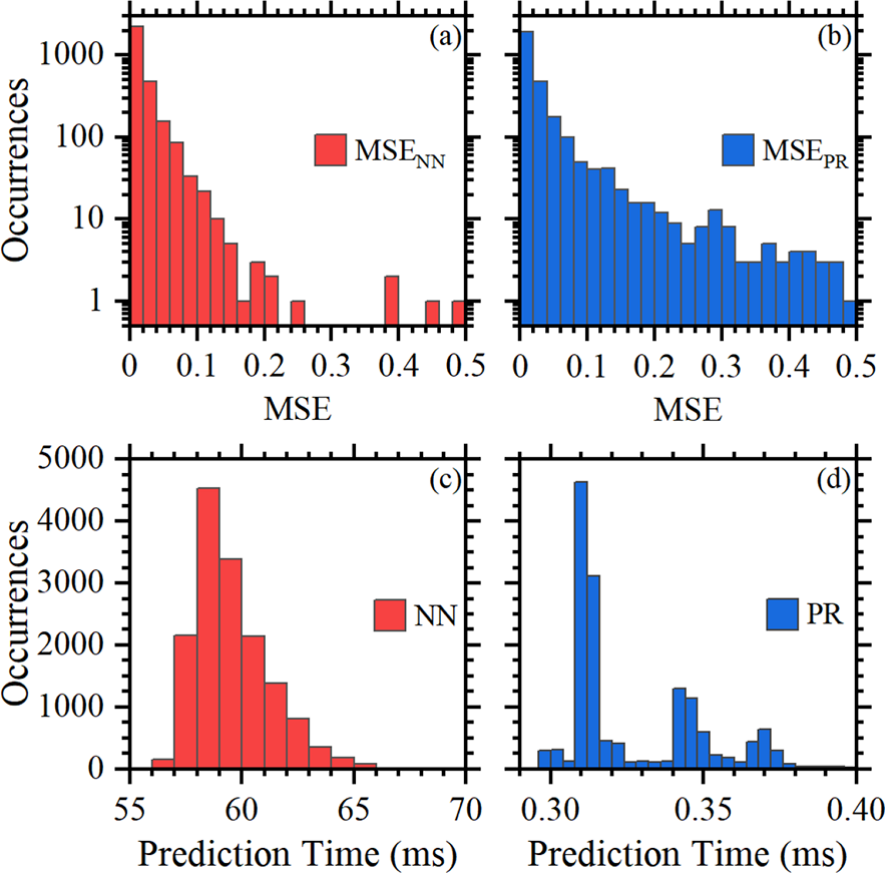
MSE histograms for the validation set using (a) NN and (b) PR, respectively, and inference time histograms for all available samples (15 213), i.e., for the training + validation sets, using the (c) NN and (d) PR methods.

Validation was also carried out using MSE, i.e., the mean of squares of the differences between FW simulations (*Y*_FW_) and predicted (*Y*_p_) values [MSE = (*Y*_FW_ – *Y*_p_)^2^], for NN (MSE_NN_) and PR (MSE_PR_) in panels a and b of Figure 5. The results indicate low MSE values, as corroborated by the larger number (2978) of data with 0 ≤ MSE ≤ 0.1 in comparison to the PR model (2736). The MSE values calculated over the entire validation data set for the PR and NN models are equal to 0.1044 and 0.009688, respectively. Nevertheless, the PR model exhibits faster prediction times than the NN model, as seen from histograms in panels c and d of Figure 5. This is due to their different computational complexities, with NNs being more computationally demanding than PR models.

The optimal NN found with NAS and used in this study consists of 3 hidden dense layers with 256, 128, and 64 nodes per layer, respectively, each utilizing ReLU activation functions. The output layer is a dense layer with 4 nodes and employs linear activation. The Adam optimizer is employed with a learning rate of 0.0001 and a batch size of 32 samples. The time complexity of the NN can be approximately represented as *O*(∑_*l* = 1_^*L*^*n*_*l*_^2^), where *L* is the number of layers and *n*_*l*_ denotes the number of nodes in the *l*th layer.

On the other hand, the PR model is a seventh-order polynomial, which has a complexity of *O*(*n*^*d*^), where *n* is the number of input parameters and *d* is the polynomial order. Despite the differences in the prediction times and efficiency, both methods deliver suitable results in intervals of milliseconds, which are several orders of magnitude shorter than the hours or days spent on conventional design/simulation tasks.

As mentioned before, the PR model uses a cross-validation approach to select the optimal polynomial degree. With the data set divided into multiple folds and the model iteratively trained and validated, cross-validation ensures that the performance of the model is evaluated on different subsets of data. This technique helps prevent overfitting by ensuring that the model generalizes well across various data splits. The MSE on the validation set for the PR model with cross-validation is equal to 0.1044. For the NN, the NAS technique was employed to find the optimal architecture. NAS systematically explored different architectures and selected the architecture with the best performance based on the evaluation metric, which, in our study, is the minimization of the MSE on the validation data set. Minimizing the MSE on the validation set mitigates the risk of overfitting. This method ensures that the chosen NN architecture is well-suited for the task without being overly complex. The MSE on the validation set for the NN with the NAS technique is equal to 0.009688. Therefore, it is reasonable to say that both models have the necessary complexity to capture the underlying behavior behind the samples and generalize effectively. Among them, the NN model demonstrates superior generalization capacity, as evidenced by its lower MSE value.

With regard to the NAS trajectory, Tables S1 and S2 of the Supporting Information present all tested configurations. Each trial trained a NN with the respective configurations for 500 epochs. The trials carried out using the NAS technique highlighted several key findings: normalization significantly improved MSE minimization; deeper and wider networks with a decreasing number of units per layer generally performed better; batch normalization and dropout showed no improvement; the Adam optimizer often outperformed SGD and was generally the best optimizer; all attempts to use the Adam optimizer with weight decay failed; and models trained with a learning rate of 0.001 generally achieved lower MSE values, indicating better performance compared to those with significantly higher or lower learning rates. The best hyperparameters for the optimal model can be seen in trial 103 of Table S2 of the Supporting Information.

**Figure 6:**
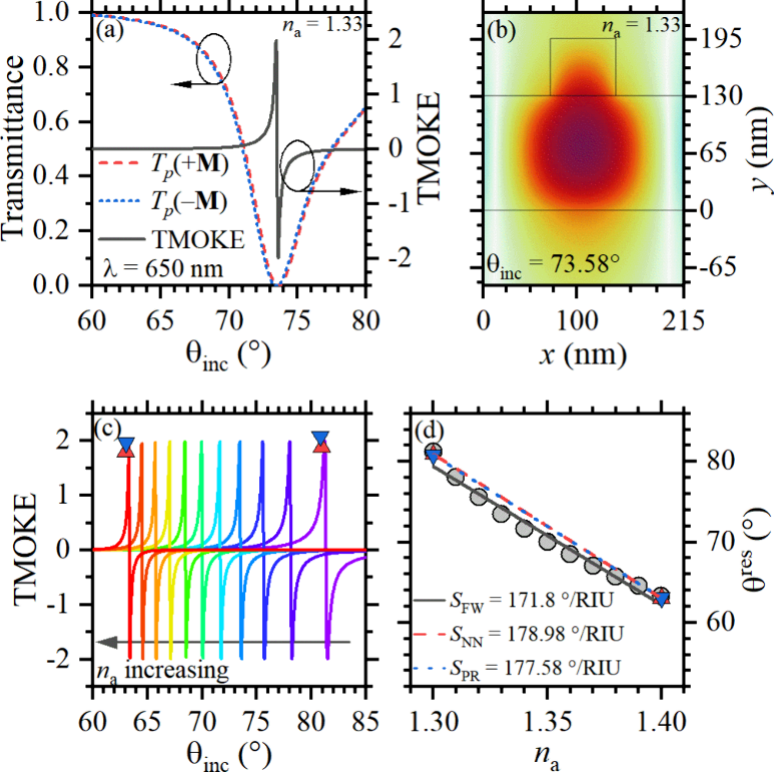
(a) Transmittances and TMOKE curves using *n*_a_ = 1.33, (b) *H*_*z*_ field coupled mode in the LiNbO_3_ waveguide, (c) TMOKE responses with corresponding *n*_a_, where the triangles are the NN (red) and PR (blue) predictions, and (d) sensitivity for FW (gray solid), NN (red dashed), and PR (blue dotted) lines.

To demonstrate the suitability of the accelerated design, we used trained NN and PR models to determine the sensitivity (*S* = |Δθ^res^/Δ*n*_a_|) of a grating design not previously encountered in the training or validation data sets. We manually chose a nanostructure with the following geometrical parameters, which we anticipate could lead to high TMOKE responses: Λ = 215 nm, *w* = 70 nm, *t*_g_ = 65 nm, *t*_LiNbO_3__ = 130 nm, and λ = 650 nm. In further work, one may conceive combining our approach with an optimization process to select high-performance nanostructures. The transmittances *T*_p_(±**M**) and TMOKE amplitudes plotted as a function of the incident angle in Figure 6a for the analyte refractive index *n*_a_ = 1.33 (conventionally used for aqueous media) and λ = 650 nm coincide with the prediction using the ML approach. The near-maximum (∼2) TMOKE amplitudes are attributed to the excitation of lateral leaky Bloch modes coupled with the guiding sublayer and the ribs of the MO grating.^11^Figure 6b shows a highly concentrated near field within the MO ribs, which is responsible for enhancing the MO activity. To quantify sensitivity, we varied the analyte refractive index and calculated the corresponding resonance shifts of the TMOKE peaks, as illustrated in Figure 6c. The TMOKE curves corresponding to *n*_a_ ranging from 1.30 to 1.40 are displayed from right to left. The normal and inverted triangles represent predicted values from the NN and PR methods, respectively, at the extreme *n*_a_ values. These predictions are consistent with full-wave electromagnetic simulation results, as noticed. The figure of merit (FoM) ranged from 672 RIU^–1^ for *n*_a_ = 1.30 to 2126 RIU^–1^ for *n*_a_ = 1.40. This variation highlights not only the high sensitivity of the designed nanostructures but also their spectral resolution capability. Such attributes are significant for biosensing at ultralow analyte concentrations. Finally, the sensitivity *S*_FW_ is determined through linear regression of TMOKE peak variations with *n*_a_, as shown by the solid gray line in Figure 6d, which is compared to sensitivities predicted from the NN (*S*_NN_) and PR (*S*_PR_) methods in the same figure. The generalization ability of the ML-accelerated design is confirmed with the results in Table 1. A maximum relative error of −8.99% was obtained in NN and PR predictions for the TMOKE peaks TMOKE_*i*_^max^ and resonant angles θ_*i*_^res^ for *n*_a_ = 1.30 (associated with *i* = 1) and *n*_a_ = 1.40 (associated with *i* = 2), along with their corresponding sensitivities.

**Table 1:** Comparison of the TMOKE^max^, θ^res^, and RIU Values Obtained in Electromagnetic Simulations (FW) to Those Predicted with the ML Models NN and PR

	TMOKE_1_^max^	TMOKE_2_^max^	θ_1_^res^ (deg)	θ_2_^res^ (deg)	*S* (deg/RIU)
FW	1.9905	1.9775	81.20	63.32	171.8
NN	1.8812	1.7998	80.93	63.03	178.98
PR	2.0870	1.9637	80.82	63.06	177.58
error (NN) (%)	–5.49	–8.99	–0.33	–0.46	+4.18
error (PR) (%)	+4.84	–0.7	–0.47	–0.41	+3.36

## Conclusion

We have devised a ML-aided approach for the accelerated design of all-dielectric magnetophotonic nanostructures. The robustness of the approach was confirmed by obtaining similar results with two distinct ML algorithms, whose predictions coincided with the results from simulations. With these ML-driven methodologies, one may circumvent the time-intensive and computationally demanding electromagnetic simulations. The ML algorithms were trained using a database of 12 170 samples containing five geometric parameters that define the nanostructure and the incident wavelength. The output was the |TMOKE^max^| and θ^res^ for two refractive indexes of the incident medium, i.e., *n*_a_ = 1.3 and 1.4, typical in biosensing in aqueous environments. It was also possible to assess sensitivity *S* = |Δθ_res_/Δ*n*_a_|, whose high value for some of the nanostructures justifies the accelerated design of all-dielectric magnetophotonic-based biosensors. It is significant that properties are predicted within milliseconds with ML algorithms, to be compared to 12 min for each full-wave electromagnetic simulation using the same computer settings. Furthermore, both training sets and algorithms can be further refined to identify nanostructure designs with optimized responses for specific working wavelengths, incidence angles, and materials tailored to the requirements of manufacturers. This versatility renders our approach valuable to experimentalists who wish to fabricate magnetophotonic nanostructures. Moreover, we should remark that the ML models presented in this work generate results based on the geometry of grating nanostructures, independent of any specific set of materials. This flexibility means that the trained algorithms can be applied to geometries that fall within the range of parameters used in the training database. However, a limitation exists: ML-based regression models typically struggle to extrapolate beyond the scope of their training data. Consequently, they do not produce reliable predictions for structures with parameters outside this predefined range. Furthermore, while the algorithms are not tied to specific materials, the resonant features of each nanograting are inherently linked to the optical properties of the materials used. This relationship suggests that generalizing the approach is feasible. By incorporation of further training, the models can learn the behavior of resonant features for different sets of materials or geometries beyond those initially considered in this study.

## Supporting Information Available

Training experimental results with the NAS technique (PDF)
