# Supplementary material for: Toward Machine-Learning-Accelerated Design of All-Dielectric Magnetophotonic Nanostructures
Source: ACS Appl Mater Interfaces. 2024 Jul 30;16(32):42828–34. doi: 10.1021/acsami.4c06740 (PMC11331439; doi:10.1021/acsami.4c06740)
Supplement: Supplementary file 1 — am4c06740_si_001.pdf [file am4c06740_si_001.pdf]

Toward Machine-Learning-Accelerated Design of All-Dielectric Magnetophotonic Nanostructures

William O. F. Carvalho ,[*,†] Marcio Tulio Aiex Taier Filho ,[‡] Osvaldo N. Oliveira Jr. ,[†] Jorge Ricardo Mejía-Salazar ,[‡] and Felipe Augusto Pereira de Figueiredo [‡]

†*Sao Carlos Institute of Physics, University of Sao Paulo, CP 369, 13560-970, São Carlos, SP, Brazil*

‡*National Institute of Telecommunications (Inatel), Santa Rita do Sapucaí, MG, 37540-000, Brazil*

E-mail: williamofcarvalho@gmail.com

Table S1: Results of training experiments with the NAS technique.

Trial	normalize	batchnorm	num layers	units 0	dropout 0	units 1	dropout 1	units 2	optimizer	lr	MSE
000	True	False	2	32	0	32	0	-	adam	0.001	1.2089
001	True	False	2	512	0	32	0	-	adam	0.001	1.1615
002	True	False	2	512	0	32	0	-	adam	1e-05	48.8944
003	True	False	2	512	0	32	0.5	-	adam	0.001	2.6397
004	True	False	2	512	0	64	0	-	adam	0.001	1.1693
005	True	True	2	512	0	32	0	-	adam	0.001	10.9579
006	False	False	2	512	0	32	0	-	adam	0.001	7.1823
007	True	False	2	512	0	32	0	-	sgd	0.001	2.6762
008	True	False	2	512	0	256	0	-	adam	0.001	0.7370
009	True	False	2	512	0	256	0.25	-	adam	0.001	1.2715
010	True	True	2	32	0.25	16	0	-	adam	0.001	39.2708
011	True	False	2	512	0	256	0	-	adam	2e-05	7.2506
012	True	False	2	512	0	256	0	-	sgd	0.001	0.4689
013	True	True	2	512	0	256	0	-	sgd	0.001	14.9669
014	True	False	3	512	0	256	0	32	sgd	0.001	0.3922
015	True	False	3	512	0	16	0	32	sgd	0.001	0.4251
016	True	False	3	512	0	256	0	32	sgd	0.001	0.3963
017	True	False	1	512	0	-	0	-	sgd	0.001	2.2797
018	True	False	3	512	0	256	0	64	sgd	0.001	0.4281
019	True	True	3	512	0	256	0	32	sgd	0.001	10.0384
020	True	False	2	512	0	256	0	-	sgd	0.001	0.4725
021	True	False	3	16	0	256	0	32	sgd	0.001	0.6647
022	True	False	3	512	0.25	256	0	32	sgd	0.001	3.8621
023	True	False	3	512	0	256	0.25	32	sgd	0.001	2.3428
024	True	False	3	512	0	64	0	32	sgd	0.001	0.3838
025	True	False	3	512	0	64	0	256	sgd	0.001	0.5019
026	False	False	3	512	0	64	0	32	sgd	0.001	49.5047
027	True	False	3	512	0	64	0	64	sgd	0.001	0.4328
028	True	True	3	512	0	64	0	32	sgd	0.001	10.0661
029	True	False	3	512	0	64	0	32	sgd	1e-05	32.5208
030	True	False	3	512	0	64	0	32	sgd	0.0001	3.0841
031	True	False	3	512	0	64	0.25	32	sgd	0.001	2.3958
032	True	False	3	16	0	64	0	32	sgd	0.001	0.8198
033	True	False	3	512	0	64	0.5	32	sgd	0.001	2.9280
034	True	False	3	512	0	64	0	32	sgd	0.001	0.4199
035	True	True	1	16	0.5	-	0	-	sgd	0.001	53.5760
036	True	False	3	512	0	1024	0	32	sgd	0.001	0.4560
037	True	False	3	512	0	64	0	32	adam	0.001	1.1140
038	True	False	2	512	0	64	0	-	sgd	0.001	2.4043
039	True	False	3	1024	0	64	0	32	sgd	0.001	0.4031
040	True	False	3	512	0	32	0	32	sgd	0.001	2.1848
041	True	False	3	512	0	512	0	32	sgd	0.001	0.4119
042	True	False	3	512	0	64	0	32	sgd	0.1	FAILED
043	True	False	3	512	0	64	0	32	sgd	2e-05	6.1893
044	True	False	3	512	0	64	0	32	adam_weight_decay	0.001	FAILED
045	True	False	3	512	0	64	0	512	sgd	0.001	0.4240
046	True	False	3	512	0	64	0	32	sgd	0.01	3.0440
047	True	False	3	128	0	64	0	32	sgd	0.001	0.5100
048	True	False	3	512	0.25	64	0	32	sgd	0.001	2.4601
049	True	False	1	512	0	-	0	-	sgd	0.001	2.4022
050	True	False	3	64	0	64	0	32	sgd	0.001	0.7000
051	True	False	3	512	0	128	0	32	sgd	0.001	0.3216
052	True	True	3	512	0	128	0	32	sgd	0.001	11.9315
053	True	False	3	512	0	128	0.5	32	sgd	0.001	2.8263
054	False	False	3	512	0	128	0	32	sgd	0.001	46.8600
055	True	False	3	512	0	128	0	32	sgd	0.1	FAILED
056	True	False	3	512	0	128	0.25	32	sgd	0.001	4.0137
057	True	True	1	512	0.25	-	0.5	-	sgd	0.01	40.6022
058	True	False	3	256	0	128	0	32	sgd	0.001	0.4754
059	True	True	1	16	0.25	-	0	-	sgd	0.001	50.4569
060	True	False	3	512	0	128	0	32	adam_weight_decay	0.001	FAILED
061	True	False	3	512	0	128	0	256	sgd	0.001	0.3132
062	True	False	3	64	0	128	0	256	sgd	0.001	0.5738
063	False	False	3	512	0	128	0	256	sgd	0.001	33.4069
064	True	False	3	32	0	128	0	256	sgd	0.001	0.6888

Table S2: Results of training experiments with the NAS technique (continued).

Trial	normalize	batchnorm	num layers	units 0	dropout 0	units 1	dropout 1	units 2	optimizer	lr	MSE
065	True	False	3	1024	0	128	0	256	sgd	0.001	0.3649
066	True	False	3	512	0	128	0.25	256	sgd	0.001	2.7191
067	True	False	3	512	0	128	0	16	sgd	0.001	0.3999
068	True	False	3	512	0	128	0	256	adam_weight_decay	0.001	FAILED
069	True	False	3	512	0.25	128	0	256	sgd	0.001	2.3298
070	True	True	1	64	0.5	-	0	-	sgd	0.001	50.2444
071	True	False	3	512	0	128	0.5	256	sgd	0.001	1.9622
072	True	False	3	512	0	512	0	256	sgd	0.001	0.4183
073	True	False	3	512	0	1024	0	256	sgd	0.001	0.3501
074	True	False	3	512	0	16	0	256	sgd	0.001	2.7460
075	True	False	3	512	0.5	128	0	256	sgd	0.001	12.9577
076	True	False	3	512	0	128	0	128	sgd	0.001	0.3566
077	True	False	3	16	0	128	0	256	sgd	0.001	0.7096
078	True	False	3	512	0	32	0	256	sgd	0.001	3.0056
079	True	False	3	512	0	128	0	256	adam	0.001	0.2877
080	True	False	2	512	0	128	0	-	adam	0.001	0.4604
081	True	False	3	512	0.25	128	0	256	adam	0.001	1.3972
082	True	False	3	512	0	128	0.25	256	adam	0.001	0.6423
083	True	False	1	512	0	-	0	-	adam	0.001	0.9500
084	False	False	3	512	0	128	0	256	adam	0.001	1.6635
085	True	True	3	512	0	128	0	256	adam	0.001	8.3242
086	True	False	3	512	0	128	0	256	adam	2e-05	1.6525
087	True	False	3	512	0	128	0.5	256	adam	0.001	1.0507
088	True	False	3	512	0	128	0	64	adam	0.001	0.8964
089	True	False	3	512	0	256	0	256	adam	0.001	0.1682
090	True	False	3	512	0	256	0.25	256	adam	0.001	0.8824
091	True	False	3	512	0	256	0	256	adam_weight_decay	0.001	FAILED
092	True	False	3	32	0	256	0	256	adam	0.001	0.8960
093	True	False	2	16	0.5	128	0	-	adam	0.001	42.5084
094	True	False	2	512	0	256	0	-	adam	0.001	0.4318
095	True	False	3	512	0.25	256	0	256	adam	0.001	0.8585
096	True	False	3	512	0	256	0.5	256	adam	0.001	0.8092
097	True	False	3	512	0	256	0	256	sgd	0.001	0.4324
098	False	False	1	1024	0.5	-	0	-	sgd	0.0001	45.0297
099	True	False	3	512	0	512	0	256	adam	0.001	0.2396
100	True	False	3	512	0.5	256	0	256	adam	0.001	2.6835
101	True	False	1	512	0	-	0	-	adam	0.001	1.0602
102	True	False	2	16	0.5	32	0	-	adam	0.001	41.6742
**103**	**True**	**False**	**3**	**256**	**0**	**128**	**0**	**64**	**adam**	**0.0001**	**0.0097**
104	True	False	3	512	0	256	0	256	adam	2e-05	0.6733
105	True	False	3	128	0	256	0	256	adam	0.001	0.3049
106	True	False	3	512	0	1024	0	256	adam	0.001	0.1694
107	True	False	3	64	0	256	0	256	adam	0.001	0.2648
108	True	False	3	512	0	256	0	256	adam	0.0001	0.4522
109	True	False	3	512	0	256	0	256	adam	0.01	0.2607
110	True	False	3	512	0	16	0	256	adam	0.001	1.1676
111	True	False	3	16	0	256	0	256	adam	0.001	0.7651
112	False	False	3	512	0	256	0	256	adam	0.001	1.8847
113	True	False	3	512	0	256	0	1024	adam	0.001	0.5583
114	True	False	3	512	0	32	0	256	adam	0.001	0.3117
115	True	True	3	512	0	256	0	256	adam	0.001	8.1012
116	True	False	3	512	0	64	0	256	adam	0.001	0.2371
117	True	False	3	512	0	256	0	256	adam	0.1	45.9184
118	True	False	3	1024	0	256	0	256	adam	0.001	0.1707
119	True	False	3	512	0	256	0	128	adam	0.001	0.2275
120	True	False	3	512	0	256	0	32	adam	0.001	0.3181
121	True	False	3	512	0	256	0	16	adam	0.001	0.3853
122	False	False	3	1024	0	512	0	1024	adam	0.001	0.5699
123	True	False	3	512	0	256	0	512	adam	0.001	0.1824
124	True	False	3	512	0	256	0	256	adam	0.001	0.6048
125	True	False	3	512	0	256	0	256	adam	1e-05	6.1504
126	True	False	1	128	0	-	0	-	adam	0.001	1.2262
127	True	False	3	512	0	256	0	64	adam	0.001	0.6045
128	True	False	1	256	0.25	-	0	-	adam	0.001	32.2839
129	False	True	2	32	0.25	32	0.25	-	adam	1e-05	52.6200
130	True	False	3	256	0	256	0	256	adam	0.001	0.2738
